# HIV Vpr induces demethylation of the SNCA antisense promoter, leading to neurocognitive impairment

**DOI:** 10.1038/s41598-026-35691-3

**Published:** 2026-01-23

**Authors:** Maryline Santerre, Ying Wang, Daniel Kalamarides, Jin Park, Lynn G. Kirby, Jeannie Chin, Jaroslav Jelinek, Natalia Shcherbik, Bassel E. Sawaya

**Affiliations:** 1 FELS Cancer Institute for Personalized Medicine, Lewis Katz School of Medicine - Temple University, 3307 North Broad Street, Philadelphia, PA 19140 USA; 2 Center for Substance Abuse Research, Lewis Katz School of Medicine - Temple University, Philadelphia, PA 19140 USA; 3 Department of Neural Sciences, Lewis Katz School of Medicine - Temple University, Philadelphia, PA 19140 USA; 4 Department of Neuroscience, Memory and Brain Research Center, Baylor College of Medicine, Houston, TX USA; 5 Coriell Institute for Medical Research, Camden, NJ USA; 6 Department for Cell and Molecular Biology, Rowan- Virtua School of Osteopathic Medicine, 2 Medical Center Drive, Stratford, NJ 08084 USA; 7 Department of Cancer and Cellular Biology, Lewis Katz School of Medicine - Temple University, Philadelphia, PA 19140 USA; 8 Present Address: Center for Discovery and Innovation, Hackensack University Medical Center, Nutley, NJ USA

**Keywords:** Neuroscience, Diseases of the nervous system, Epigenetics in the nervous system

## Abstract

Human immunodeficiency virus type 1 (HIV-1) alpha-synuclein (α-Syn) aggregation is a hallmark of neurodegenerative diseases. Accumulation and aggregation of α-Syn are often observed in individuals with HIV-1 cognitive impairments. The direct mechanistic link between α-Syn dysregulation and HIV-associated neurocognitive disorders (HAND) remains unclear. Emerging evidence suggests that epigenetic changes, particularly deoxyribonucleic acid (DNA) demethylation, influence α-Syn regulation. We show that the HIV-1 protein viral protein R (Vpr) demethylates the antisense promoter within intron 1 of the alpha-synuclein gene (*SNCA*), potentially contributing to increased α-Syn expression. Elevated α-Syn promotes aggregation, causing synaptic dysfunction and impaired mitochondrial transport. These processes contribute to the development of HAND. Furthermore, we find that Vpr’s activation of the SNCA antisense promoter depends on demethylation; dimethyloxaloylglycine (DMOG), a ten-eleven translocation (Tet) inhibitor, reverses this demethylation and reduces Vpr-induced SNCA antisense activation. Our findings suggest that α-Syn dysregulation plays a role in cognitive decline among people living with HIV and that targeting α-Syn regulatory pathways could help reduce HIV-related neurodegeneration. To our knowledge, this is the first study to show that an HIV protein epigenetically activates the SNCA antisense promoter, linking viral infection to α-synuclein deregulation. Future research should explore how SNCA antisense promoter demethylation leads to neuronal dysfunction and examine the broader impact of α-Syn dysregulation on neuronal health.

**Supplementary Information:**

The online version contains supplementary material available at 10.1038/s41598-026-35691-3.

## Introduction

HIV-associated neurocognitive disorders (HAND) still pose a major clinical challenge despite the widespread use of combination antiretroviral therapy (cART). HAND encompasses a variety of cognitive, motor, and behavioral impairments caused by ongoing inflammation and neurodegeneration in the brain due to HIV infection^1^. A subset of individuals with HAND exhibit Parkinson-like motor symptoms, such as tremors, bradykinesia, rigidity, and postural instability, which closely resemble features observed in Parkinson’s disease (PD)^2–7^.

Four interconnected mechanistic pathways drive HAND-related neurodegeneration. First, HIV infects glial cells, causing neuronal damage in motor control regions such as the basal ganglia and promoting local chronic inflammation. Second, ongoing immune activation leads to widespread chronic inflammation, even with cART treatment. Third, toxic viral proteins, including the HIV-1 transactivator of transcription (Tat) and Vpr, disrupt cellular homeostasis and exacerbate neurotoxicity, in part by modulating inflammatory processes. Fourth, these viral proteins promote the buildup of alpha-synuclein (α-Syn), linking HAND-related degeneration to Parkinson-like symptoms through neuronal dysfunction^8^. Although cART reduces some symptoms, neurodegeneration often continues, highlighting the need to target these interconnected mechanisms beyond just suppressing the virus^9,10^.

HIV-1 viral protein R (Vpr) is a 15-kDa multifunctional protein that supports viral nuclear import, causes G2-phase arrest, and regulates the HIV-1 promoter^11^. Vpr also interacts with the host’s epigenetic machinery and promotes DNA demethylation^12,13^. Previous research has shown Vpr’s effects on cellular pathways; however, the mechanisms by which Vpr interacts with host epigenetic systems to control alpha-synuclein gene (*SNCA*) expression remain poorly understood. The direct link between Vpr-induced epigenetic changes and α-Syn accumulation, as well as their effects on neuronal function, is a significant knowledge gap. The present study aims to fill this gap. Recent data show that Vpr reactivates the SNCA gene’s antisense promoter in intron 1, thereby increasing α-Syn transcription. This buildup alters lysosomal pH, hampers autophagy, and promotes α-Syn aggregation, contributing to neuronal dysfunction and HAND pathogenesis^14,15^.

Alpha-synuclein (α-Syn) is a 14-kDa protein located at presynaptic terminals and is essential for neurotransmitter release and synaptic plasticity^16,17^. Genetic and epigenetic modifications, such as SNCA antisense promoter demethylation, increase α-Syn levels and promote its aggregation^18,19^. Aggregated α-Syn impairs mitochondrial and lysosomal functions, disrupts synaptic communication, and elevates oxidative stress, leading to neuronal loss. Additionally, α-Syn activates microglia and astrocytes, prompting the release of pro-inflammatory cytokines that exacerbate neuroinflammation and neuronal injury^14,15^.

The antisense promoter in intron 1 of the SNCA gene plays a crucial role in regulating α-Syn expression. It modulates transcriptional activity through antisense ribonucleic acid (RNA) transcripts, which influence overall SNCA messenger RNA (mRNA) and protein levels. Epigenetic modifications, such as promoter DNA methylation, can alter α-Syn expression, connecting this promoter to HAND and other neurodegenerative diseases^20^.

This study demonstrates that HIV-1 Vpr reactivates the SNCA antisense promoter, leading to α-Syn aggregation and neurotoxicity by impacting inflammation, autophagy, and synaptic function. These findings clarify how HIV-1 promotes neurodegeneration related to HAND and emphasize the potential of targeting α-Syn regulation as a therapeutic strategy for people living with HIV (PLWH).

## Methods

### Cell culture

SH-SY5Y neuroblastoma cells were obtained from the American Type Culture Collection (ATCC) (CRL-2266) and cultured as described previously^21^. Cells were differentiated with 10 μM retinoic acid for at least 4 days before treatment and subsequent experiments.

Primary neuronal cultures were prepared as previously described with slight modifications. Briefly, hippocampal tissues were collected from 17–18-day-old embryonic C57BL/6 mice (Taconic Farms- Hudson, NY, USA), digested in 0.125% trypsin for 30 min, and then washed twice in Hank’s Balanced Salt Solution (HBSS) (Corning Cellgro- Corning, NY, USA). The digested tissue was then triturated and dissociated into single cells in seeding medium (Dulbecco’s Modified Eagle Medium [DMEM]) containing 4.5 g/L glucose, 10% fetal bovine serum (FBS), 1 × GlutaMAX, 1 × nonessential amino acids, 100 IU/ml penicillin and streptomycin; Invitrogen—Carlsbad, CA, USA). Cells in suspension were centrifuged at 150 × g for 10 min at room temperature (20–22 °C), and the pellet was gently resuspended in the seeding medium. The pellet was then filtered through a 400-mesh screen to remove undispersed tissue before being seeded onto plates.

### Recombinant proteins and treatment

HIV-1 Vpr (100 ng/ml), produced in SF9 insect cells (BioBasic Canada Inc.- Markham, ON, Canada), was purified to over 95% purity as confirmed by sodium dodecyl sulfate–polyacrylamide gel electrophoresis (SDS-PAGE) and tested for biological activity through cell cycle arrest assays before use. The recombinant protein was applied to the cells at 7 nM (100 ng/ml) for all experiments unless otherwise noted.

### Chemical reagents

Cells were treated with decitabine (5-Aza-2'-deoxycytidine, 5-Aza-dC) (5 μM, purchased from Sigma-Aldrich- St. Louis, MO, USA), a United States Food and Drug Administration (FDA)-approved drug that inhibits DNA methylation, for 24 and 48 h before adding Vpr protein. Additionally, cells were treated with 0.5 or 1.0 mM dimethyloxaloylglycine (DMOG) for 48 h prior to the addition of Vpr protein.

### RNA extraction

Total RNA was isolated from the sample using the SurePrep^™^ TrueTotal^™^ RNA purification kit (BP280050; Fisher Scientific, Waltham, MA, USA) according to the manufacturer’s instructions. Briefly, cells were lysed in TrueTotal™ lysis buffer, and samples were centrifuged at 12,000 × g for 10 min at 4 °C to pellet cellular debris. The supernatant was transferred to a clean tube, and RNA was precipitated with isopropanol and collected by centrifugation at 12,000 × g for 10 min at 4 °C. The RNA pellet was washed twice with 75% ethanol (centrifugation at 7500 × g for 5 min at 4 °C), air-dried at room temperature, and resuspended in nuclease-free water. A NanoDrop spectrophotometer (Thermo Fisher Scientific, Waltham, MA, USA) was used to assess the purity and concentration of the extracted RNA, with A260/A280 ratios between 1.8 and 2.0 considered acceptable.

### Stereotaxic surgery and spatial memory testing

C57BL/6 J male mice (8–10 weeks old) (purchased from the Jackson Lab, Bar Harbor, ME, USA) were housed individually with free access to food and water before and after stereotaxic surgery. Under isoflurane anesthesia, mice received bilateral intrahippocampal injections (1 μl per site) of either saline or Vpr (100 ng/μl) at rostral and caudal coordinates, totaling four injections per mouse. Intraperitoneal saline was given immediately and again 36 h post-surgery. Seventy-two hours after injection, spatial memory was tested using the object location memory test. In this test, mice explored two identical objects in an arena during three training trials, with exploration time recorded. After 24 h, one object was moved, and mice were tested for 3 min; exploration time was measured to evaluate spatial memory. Greater exploration of the displaced object indicated intact spatial memory. All procedures were approved by the Baylor College of Medicine Institutional Animal Care and Use Committee (IACUC protocol #AN-6943, approved on 09/2024 – PI: Dr. Jeannie Chin).

### Real-time PCR

Total RNA was extracted using TRIzol reagent (Invitrogen, Carlsbad, CA, USA) according to the manufacturer’s protocol, and reverse-transcribed into complementary DNA (cDNA) using the SuperScript VILO cDNA Synthesis Kit (Invitrogen). Real-time polymerase chain reaction (PCR) was performed with the SYBR Premix Real-Time PCR Kit (Roche, Basel, Switzerland) according to the manufacturer’s instructions. mRNA levels were normalized to β-actin and expressed as 2^-ΔΔCt^.

The primer sequences are as follows:

SNCA forward: 5′-ggctttgtcaagaaggaccag-3′, reverse: 5′-cctctgaaggcatttcataagcc-3′.

β-actin forward: 5′-ggctgtattcccctccatcg-3′, reverse: 5′-cgtcccagttggtaacaatgcc-3′.

For experiments with DMOG, we used the following primers:

SNCA forward: 5'-ccagttgggcaagaatgaagaa-3', reverse: 5'-cttgatacccttcctcagaaggc-3;

SNCA-AS forward: 5'-gcccaagaaataacacgcaac-3', and reverse: 5'-caatgctccttgagctttcc-3'.

Glyceraldehyde-3-phosphate dehydrogenase (GAPDH) forward: 5'-tcgacagtcagccgcatcttcttt-3', and reverse: 5’-accaaatccgttgactccgacctt-3'.

### Bisulfite sequencing

Bisulfite treatment of genomic DNA was performed using a Zymo bisulfite conversion kit (Zymo Research, Irvine, CA, USA). Genomic DNA was extracted from the brains of HIV patients or age-matched healthy controls (IBC #4195, approved on 2/24/2025). Bisulfite-converted DNA was amplified with primers targeting specific genomic regions. PCR products were cloned into the pCR4 TOPO vector according to the manufacturer’s instructions. Colony PCR was conducted with a FailSafe colony PCR kit, and plasmids were purified using the Promega Wizard Plus SV Minipreps kit (Promega, Madison, WI, USA). At least 20 colonies were sent for sequencing. The sequencing results were analyzed using the QUMA online bioinformatics tool with the following parameters: a minimum sequence identity of 90%, a minimum conversion rate of 95%. Cytosine-phosphate-guanine dinucleotide (CpG) sites were considered methylated when methylation was detected in more than 50% of reads.

Case-level metadata for all human samples (de-identified ID, age, sex, HIV/antiretroviral therapy (ART) group, and repository neuropathology summary) are provided in Supplementary Table S1; no additional data on comorbidities, ART regimens, or viral loads were available from the repository.

### Slice preparation, electrophysiology, and data analysis

Hippocampal slices were prepared from adult mice (both males and females) obtained from Jackson Laboratory (Bar Harbor, ME, USA), and electrophysiological recordings were performed as previously described^22^. Animals were housed under standard conditions with unrestricted access to food and water. All procedures conformed to Temple University Institutional Animal Care and Use Committee (IACUC) (protocols # 4571, approved on 04/03/2025, and # 4862, approved on 04/03/2025 – PI: Dr. Lynn G Kirby) animal care guidelines and the National Research Council *Guide for the Care and Use of Laboratory Animals*. Briefly, brains were extracted and placed in artificial cerebrospinal fluid (aCSF) prepared with sucrose (248 mM) instead of sodium chloride (NaCl). Using a vibratome (Vibratome 3000 plus; Vibratome, Bannockburn, IL, USA), transverse hippocampal sections (400 µm thick) were sliced. The slices were incubated for three hours in aCSF (in mM: 124 NaCl, 2.5 potassium chloride (KCl), 2 sodium phosphate monobasic (NaH_2_PO_4_), calcium chloride (CaCl_2_), 2 magnesium sulfate (MgSO_4_), 10 dextrose, and 26 sodium bicarbonate (NaHCO_3_)) or aCSF containing 200 ng/ml Vpr at room temperature, bubbled with a gas mixture of 95% oxygen (O2) and 5% carbon dioxide (CO2). Subsequently, the slices were transferred to recording chambers (Warner Instruments, Hamden, CT, USA) and perfused with aCSF at a rate of 1.5–2.0 mL/min, bubbled with 95% O_2_/5% CO_2,_ and maintained at 32–34 °C using an inline solution heater (TC-324; Warner Instruments). Field excitatory postsynaptic potentials (fEPSPs) were recorded in the dorsal hippocampus using an extracellular glass pipette (3–5 MΩ) filled with aCSF. Recording electrodes were placed in the stratum radiatum of area CA1, with bipolar tungsten stimulating electrodes positioned 200–300 μm from the pipette along the Schaffer collateral fibers in the stratum radiatum. An input–output curve was initially generated (stimulating from 0 to 300 microampere (μA) in 20 μA steps), and a stimulus intensity was chosen that elicited approximately one-third of the maximum fEPSP amplitude. Baseline fEPSPs were recorded in response to stimuli delivered every 30 s for 20 min prior to tetanization. Slices with unstable baselines, specifically, those where the normalized rise/slope decreased by 20–50 millivolts per millisecond (mV/msec) over roughly 10 min, were excluded. Long-term potentiation (LTP) was induced by four trains of 100-Hz stimulation separated by 20 s. Post-tetanus fEPSPs were recorded at 30-s intervals for 90 min following tetanization. The rise/slope of the fEPSP (mV/ms) between 30 and 90% was measured using Clampfit 10.3 (Molecular Devices, Sunnyvale, CA, USA) and normalized to the mean baseline. Data are expressed as the percentage change from the baseline fEPSP slope. Recordings and data analysis were performed by investigators blinded to treatment conditions. Fluorescence intensity was measured using ImageJ software (National Institutes of Health, Bethesda, MD, USA), with background subtraction and normalization to cell count based on 4’,6-diamidino-2-phenylindole (DAPI)-positive nuclei.

### Immunofluorescence of SH-SY5Y cells with SNCA

After 3 weeks of treatment with 100 ng/ml of recombinant HIV-1 Vpr protein during retinoic acid differentiation, the medium was removed, and cells were fixed in 4% formaldehyde (Sigma-Aldrich, 252,549) in phosphate-buffered saline (PBS). SH-SY5Y cells (ATCC^®^ CRL-2266^™^) were stained for SNCA/α-synuclein. Cells were permeabilized with 0.2% Triton X-100 (Sigma, St. Louis, MO, USA, T8787) in PBS for 5 min. Primary rabbit anti-SNCA/α-synuclein antibodies (1:500; Santa Cruz Biotechnology, Dallas, TX, USA, sc-7011) were diluted in 3% (w/v) bovine serum albumin (Sigma-Aldrich, A3059) in PBS and incubated overnight at 4 °C. Donkey anti-Rabbit IgG (H + L) Highly Cross-Adsorbed Secondary Antibody, Alexa Fluor 647 (Invitrogen, A-31573), was used at 1:1000 dilution. Cells were mounted on glass slides and imaged using a 60 × oil-immersion objective on a confocal microscope (Leica DMI 4000B, Leica Microsystems, Wetzlar, Germany). For immunofluorescence analysis, images were quantified using ImageJ to measure the mean fluorescence intensity per cell (arbitrary units) from at least 5 randomly selected fields per condition (~ 100 cells total). The results were presented as bar graphs.

### Statistical analysis

All experiments were conducted at least three times with independent batches of cell cultures. Data are presented as means ± standard error of the mean (SEM). Student’s t-test was used for two-group comparisons. Multiple comparisons were analyzed by one-way analysis of variance (ANOVA) followed by the least significant difference (LSD) test. Statistical analyses were performed using GraphPad Prism software (GraphPad Software, San Diego, CA, USA). *p < 0.05, **p < 0.01 versus the mock group; or versus rVpr-treated group.

### Software and data analysis

Image quantification was performed using ImageJ software (National Institutes of Health, Bethesda, MD, USA; https://imagej.nih.gov/ij/). Electrophysiological data were analyzed using Clampfit software (Molecular Devices, Sunnyvale, CA, USA). Bisulfite sequencing analysis was conducted using the QUMA online bioinformatics tool (http://quma.cdb.riken.jp/). Statistical analyses were performed using GraphPad Prism software (GraphPad Software, San Diego, CA, USA; https://www.graphpad.com).

### Humane endpoints and euthanasia

Mice were euthanized using gradual-fill CO₂ inhalation (20–30% chamber volume displacement per minute) followed immediately by decapitation. For electrophysiology experiments, animals were deeply anesthetized with isoflurane (5% induction, 3–4% maintenance) prior to decapitation, and brains were rapidly extracted for acute slice preparation in accordance with Temple University protocols. For behavioral studies, mice were euthanized by intraperitoneal (IP) injection of a commercial euthanasia solution, followed by bilateral thoracotomy and transcardial perfusion with saline or phosphate-buffered saline, in accordance with Baylor College of Medicine–approved protocols. All euthanasia procedures were performed by trained personnel in compliance with IACUC-approved protocols and American Veterinary Medical Association (AVMA) guidelines.

### Human ethical statement

All experiments involving human brain tissue samples were conducted in accordance with the guidelines set by the National Institutes of Health (NIH). De-identified human tissue samples—limited to information on gender, age, and HIV status—were obtained from the National NeuroAIDS Tissue Consortium (NNTC) and approved by the Institutional Biosafety Committee (IBC) (IBC #11,182, approved on 03/12/2024 – PI: Dr. Bassel E Sawaya) and the Institutional Review Board (IRB) (protocol #4195, approved on 2/24/2025 – PI: Dr. Bassel E Sawaya) at Temple University. For details on the samples used, see Table S1. Consent from guardians was not required because the samples were de-identified prior to receipt.

### Animal ethical statement

All experiments involving mouse samples were performed in accordance with the guidelines set by the National Institutes of Health (NIH). All animal protocols received approval from the Institutional Animal Care and Use Committee (IACUC) at either Temple University (IACUC # 5195, approved on 05/05/2025 – PI: Dr. Bassel E Sawaya), (IACUC # 4571, approved on 04/03/2025 and IACUC # 4862, approved on 04/03/2025 – PI: Dr. Lynn G Kirby) or Baylor College of Medicine (protocol #AN-6943, approved on 09/2024 – PI: Dr. Jeannie Chin). Animal studies adhered to the Animal Research: Reporting of In Vivo Experiments (ARRIVE) procedures to ensure ethical standards and research reproducibility.

## Results

### Alpha-synuclein accumulation: a shared mechanism in aging and neurodegeneration models

**Fig. 1:**
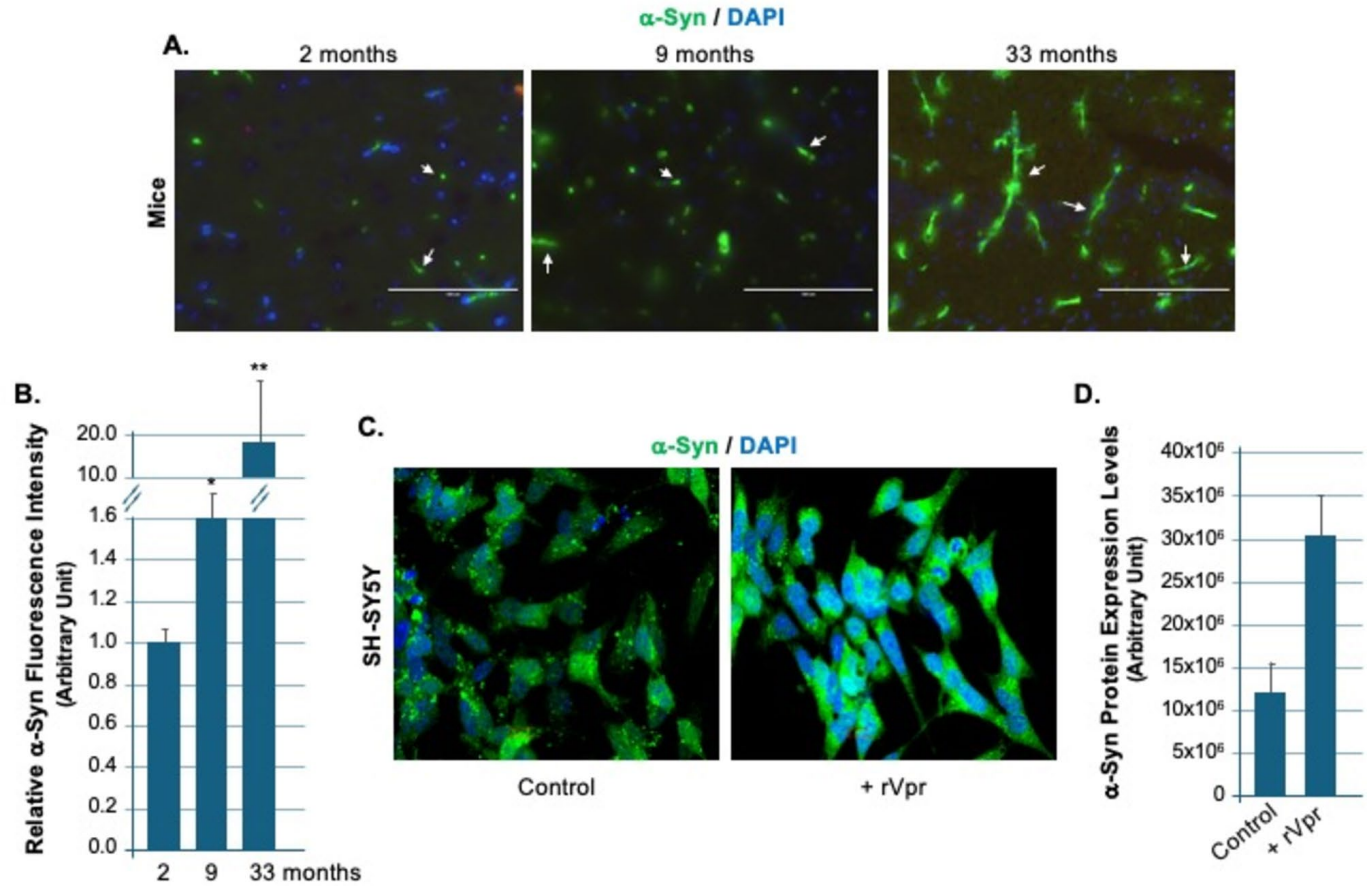
Accumulation of α-Synuclein in Aged Mice and Vpr-Treated SH-SY5Y Cells. **A** Immunofluorescence staining shows α-Syn (green) and nuclei (DAPI, blue) in brain tissue from mice aged 2, 9, and 33 months. α-Syn accumulation increases with age. Scale bars are 50 μm. **B** Quantification of Panel A shows α-synuclein fluorescence intensity for all age groups (n = 3 each). Asterisks indicate significant differences by one-way ANOVA with post-hoc testing (*p < 0.05; *p < 0.01). **C** Immunofluorescence staining reveals α-Syn (green) and nuclei (DAPI, blue) in SH-SY5Y cells. **D** A bar graph compares α-Syn expression levels in Vpr-treated and control cells. The observed increase in α-Syn in Vpr-treated cells relative to controls may indicate that Vpr influences mechanisms underlying α-Syn accumulation. Scale bars are 50 μm.

Previous studies showed increased α-Syn levels in human brains infected with HIV, monkey brains infected with simian immunodeficiency virus (SIV), and in cell line models^14^. In this study, endogenous α-Syn levels were measured in mice aged 2, 9, and 33 months. The results revealed age-dependent accumulation. Representative immunofluorescence staining is shown in Fig. 1A, and quantification is shown in Fig. 1B. Quantification of fluorescence intensity indicated a significant increase in α-Syn levels with age. Mice aged 33 months had approximately 3.5 times higher expression than 2-month-old mice (p < 0.01). These findings support the hypothesis that α-Syn accumulation adversely affects aging, memory, and movement.

To further explore these observations, Vpr-treated SH-SY5Y cells were analyzed. A similar increase in α-Syn levels was observed. Representative images are shown in Fig. 1C, and quantification is shown in Fig. 1D. This aligns with previous findings in human and monkey models and facilitates investigation of the molecular mechanisms underlying α-Syn accumulation. Although the models differ, they complement each other and collectively support the role of α-Syn in aging and neurodegeneration.

### HIV-1 Vpr alters epigenetic regulation at the SNCA antisense promoter

**Fig. 2:**
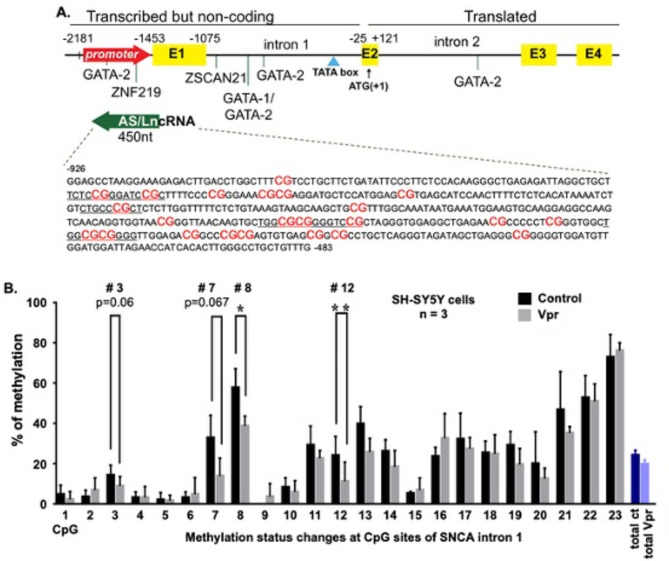
HIV-1 Vpr Reduces CpG Methylation at the SNCA Antisense Promoter. **A** Structure of the SNCA gene, highlighting coding exons (yellow boxes) and the antisense promoter (green arrow) within intron 1. Transcription factors (GATA-1 and 2, ZSCAN21, ZNF219), regulatory elements (TATA box), and the 450 nt-long antisense RNA are indicated. The SNCA antisense promoter sequence, with 23 analyzed CpG sites (red), is also shown. **B** Methylation levels at the SNCA antisense promoter in Vpr-treated SH-SY5Y cells reveal significant demethylation at CpG sites 3, 7, 8, and 12 (*p < 0.05, **p < 0.01). Data are presented as the mean ± SD from n = 3 replicates. Statistical significance was determined by one-way ANOVA.

Previous studies have demonstrated α-Syn aggregation in Vpr-treated cells^14,15^. Data from Fig. 1 support further investigation into whether Vpr affects α-Syn at the transcriptional level through epigenetic regulation of the SNCA antisense promoter. Several transcription factors regulate the SNCA gene. A unique regulatory element is the antisense promoter, which is located within intron 1 of the gene. A schematic representation of the SNCA gene structure is shown in Fig. 2A. This 450-nucleotide region contains 23 CpG sites. Some studies describe this region as a long non-coding RNA (lncRNA) that influences SNCA gene expression.

To further investigate the underlying mechanisms, the study focused on the epigenetic regulation of α-Syn expression. The SNCA antisense promoter is a crucial regulatory region that becomes active upon demethylation. This activation influences SNCA gene expression. Significant demethylation was observed at CpG sites 8 and 12 within the promoter, suggesting a key role in epigenetic regulation of SNCA expression in response to Vpr treatment. Additionally, CpG sites 3, 7, 8, and 12 showed demethylation, although to a lesser extent, and may still contribute to changes in gene expression. Methylation analysis is shown in Fig. 2B (*p < 0.05, **p < 0.01). These findings highlight the importance of these CpG sites in modulating SNCA expression. This prompted further exploration of Vpr’s impact on α-synuclein levels. The results suggest that Vpr directly induces epigenetic modifications within the SNCA antisense promoter. This indicates a potential mechanism by which Vpr regulates α-Syn expression and contributes to its pathological aggregation.

### Differential expression of α-synuclein and its antisense promoter in response to HIV-1 Vpr

**Fig. 3:**
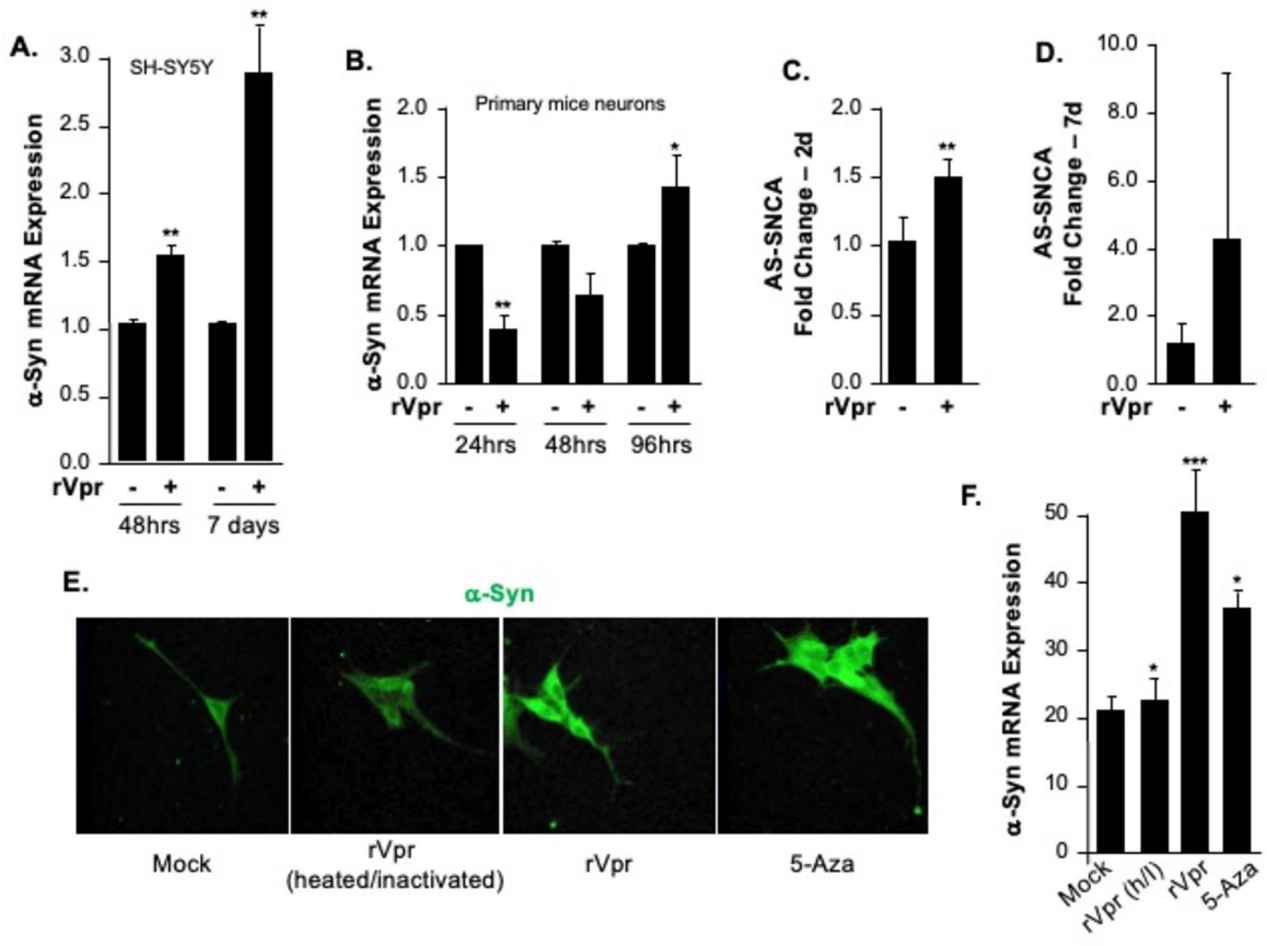
Differential expression of α-Syn and its antisense promoter in response to HIV or Vpr. **A**, **B** qPCR shows α-Syn mRNA in SH-SY5Y cells (**A**) and primary mouse neurons (**B**) after rVpr (7 nM) for various times. **C**, **D** Antisense SNCA promoter activity increases in SH-SY5Y cells at 48 h and 7 days after Vpr treatment. Data are mean ± SEM. Statistical significance was tested (e.g., ANOVA, t-test). **E** Images show increased α-Syn in neuronal cells treated with 7 nM Vpr. Control and heat-inactivated Vpr groups show reduced levels. The methylation inhibitor 5-Aza-dC reduces α-Syn, indicating that Vpr regulates α-Syn via epigenetic mechanisms. The ***F.*** Bar graph quantifies fluorescence from panel E, with mean ± SEM (n = 3). Active rVpr raises α-Syn over all groups (***p < 0.001). 5-Aza partially reduces α-Syn levels vs active rVpr (*p < 0.05). Mock and rVpr (inactivated) maintain low baseline fluorescence. One-way ANOVA with Tukey’s post hoc test was used.

Building on the methylation analysis presented in Fig. 2, the current study investigated the functional effects of Vpr-induced demethylation on α-Syn expression and the activity of the SNCA antisense promoter. As shown in Fig. 3A and B, Vpr treatment caused a significant, time-dependent increase in α-Syn expression in both SH-SY5Y cells and primary mouse neurons. This confirms that Vpr promotes α-Syn overexpression at the mRNA level. The increase in α-Syn expression was closely associated with the upregulation of the SNCA antisense promoter. This is depicted in Fig. 3C and D, suggesting that transcriptional regulation is linked to Vpr activity. The results indicate that Vpr-induced demethylation at CpG sites 8 and 12 (as shown in Fig. 2) likely contributes to increased transcriptional activation of the SNCA gene. These findings further support the idea that Vpr plays a key role in regulating α-Syn expression and its related pathological aggregation.

### Epigenetic mechanisms drive α-Syn overexpression

To explore the link between DNA methylation and α-Syn accumulation, the FDA-approved DNA methylation inhibitor 5-Aza-dC (Aza) was used to promote DNA demethylation. Treatment with 5-Aza-dC was compared to a negative control using heat-inactivated Vpr. Notably, α-Syn accumulation increased in cells treated with 5-Aza-dC. Conversely, cells exposed to active Vpr exhibited significantly higher α-Syn levels than cells in both control groups and those treated with heat-inactivated Vpr. Representative images are shown in Fig. 3E, with quantification presented in Fig. 3F. These findings highlight the importance of epigenetic regulation, especially CpG demethylation, in Vpr-mediated α-Syn overexpression.

### Demethylation of SNCA Intron 1 in human brain tissues

**Fig. 4:**
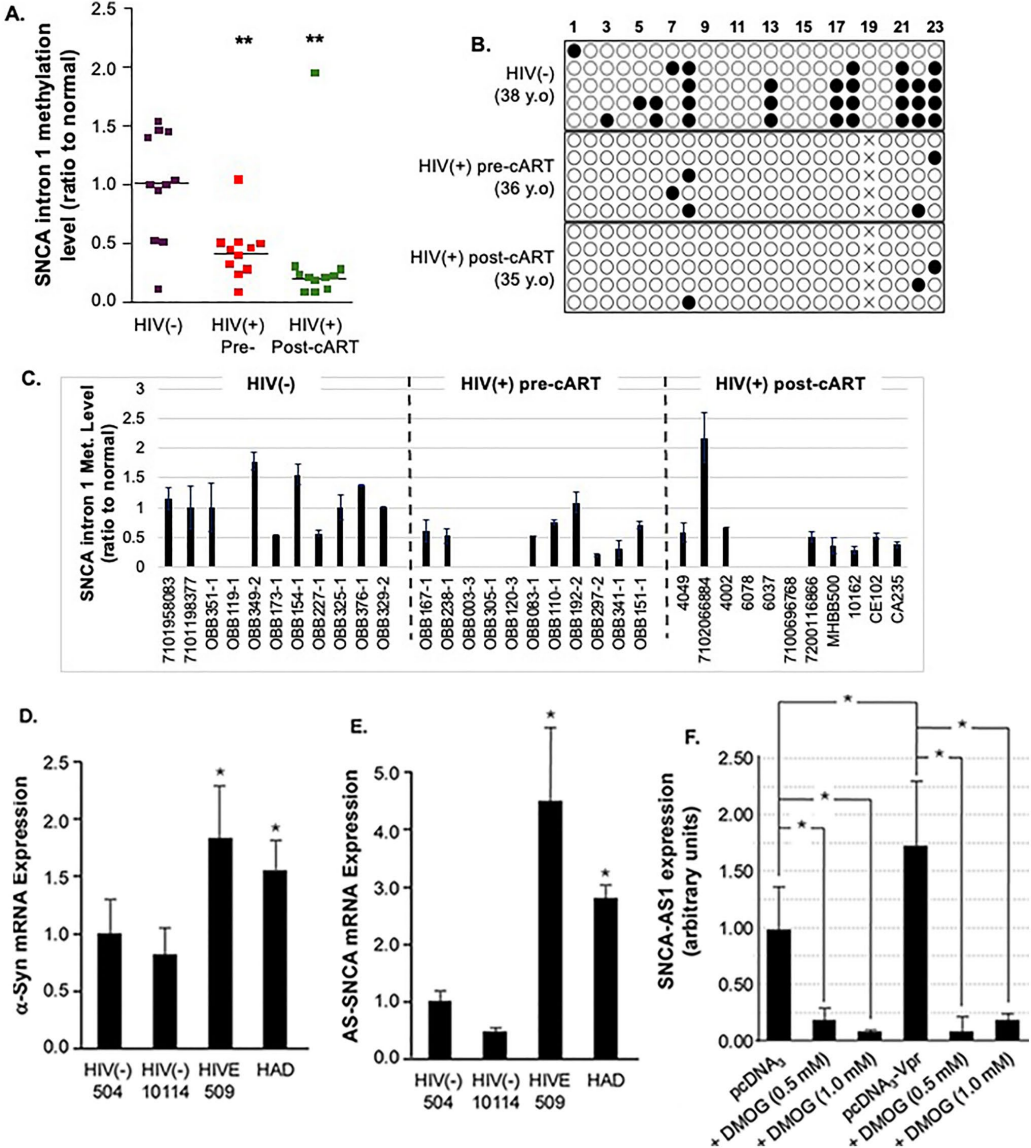
Demethylation of SNCA Intron 1 in human brain tissues. **A** A scatter plot displays SNCA intron 1 methylation levels in HIV-positive pre- and post-cART groups and HIV-negative controls. HIV-positive groups show significantly reduced methylation (p < 0.05, one-way ANOVA with Tukey’s post-hoc test, n = 13 per group). **B** Representative bisulfite sequencing profiles of SNCA intron 1 in HIV-negative, HIV-positive pre-, and post-cART individuals. Open circles indicate unmethylated CpG sites, while filled circles indicate methylated CpG sites. The HIV-positive sample exhibits decreased methylation. **C** A bar graph shows SNCA intron 1 methylation levels (relative to normal) in samples from the NNTC cohort. HIV-positive pre- and post-ART samples have significantly lower methylation than HIV-negative controls (p < 0.01, Kruskal–Wallis test with Dunn’s multiple comparisons, n = 13 per group). **D, E** Increased α-Syn (*D*) and SNCA antisense promoter (*E*) mRNA expression are observed in brain tissues of people living with HIV (PLWH). Expression is higher in patients with HIVE and HAD than in controls (p < 0.05, one-way ANOVA with Tukey’s post hoc, n = 3 per group). **F** SNCA-AS1 expression was measured by qPCR in SH-SY5Y cells transfected with pcDNA3 or pcDNA3-Vpr plasmids and treated with DMOG (0.5 or 1.0 mM). Data are shown as mean ± SEM. Two-way ANOVA determined statistical significance with Tukey’s post-hoc test (*p < 0.05, p < 0.01, n = 3 per condition). Cohort details (**A**–**E**): de-identified male donors from the NNTC. Supplementary Table S1 provides additional information, including case ID, age, HIV/ART group, and repository neuropathology summaries. Only age, sex, HIV/ART status, and brief neuropathology data were available. Data on broader comorbidity, ART regimen, and viral load were unavailable.

To extend these findings to human samples, the methylation status of SNCA intron 1 was examined in brain tissues from HIV-positive individuals both before and after cART. Comparisons were made to HIV-negative controls. Both HIV-positive groups showed significantly lower methylation levels compared to controls. Methylation levels are shown in Fig. 4A. The decrease in methylation was consistent across pre- and post-cART HIV-positive groups. This indicates that HIV infection, rather than cART treatment, is the primary factor behind these epigenetic changes. Bisulfite sequencing profiles showed clear demethylation in HIV-positive samples, with a notable loss of methylation marks relative to controls. Representative bisulfite sequencing profiles are shown in Fig. 4B. Quantitative analysis confirmed these reductions, with statistical significance indicating HIV-related changes in the DNA methylation landscape of SNCA intron 1. Quantification is shown in Fig. 4C. These findings suggest a lasting HIV-mediated epigenetic modification of SNCA intron 1 that occurs independently of cART. This alteration could contribute to the development of HIV-associated neurodegenerative disorders.

To determine whether these epigenetic changes are associated with changes in gene expression, levels of α-Syn and SNCA antisense promoter mRNA were measured in brain tissues from people living with HIV (PLWH). The analysis was divided based on HIV-related conditions like HIV encephalitis (HIVE) and HIV-associated dementia (HAD). α-Syn levels are shown in Fig. 4D, and SNCA antisense promoter levels are shown in Fig. 4E. Both α-Syn and SNCA antisense promoter levels were significantly higher in individuals with HIVE and HAD compared to HIV-negative controls. These findings indicate that HIV infection, along with persistent epigenetic modifications such as demethylation of SNCA intron 1, leads to increased α-Syn expression. This increase may play a role in the neuropathology seen in HIV-related neurodegenerative diseases. The observed link between increased α-Syn expression and HIV-related neuropathology further supports the idea that HIV-induced gene regulation changes contribute to cognitive and motor impairments in PLWH.

### Confirmation of Vpr-mediated demethylation pathway

To further verify the role of the demethylation pathway in Vpr-induced enhancement of α-Syn accumulation and aggregation, cells were treated with DMOG (0.5 and 1.0 mM) for 48 h. DMOG is a non-specific inhibitor of 2-oxoglutarate-dependent dioxygenases and blocks Tet enzymes, thus preventing DNA demethylation. Vpr is believed to promote demethylation of CpG sites within the antisense promoter. Therefore, DMOG treatment is expected to inhibit this process. As a result, DMOG prevented demethylation of the antisense promoter and reduced Vpr-induced activation of the antisense transcript. Antisense transcript levels are shown in Fig. 4F.

### HIV-1 Vpr causes synaptic and memory problems in a mouse model

**Fig. 5:**
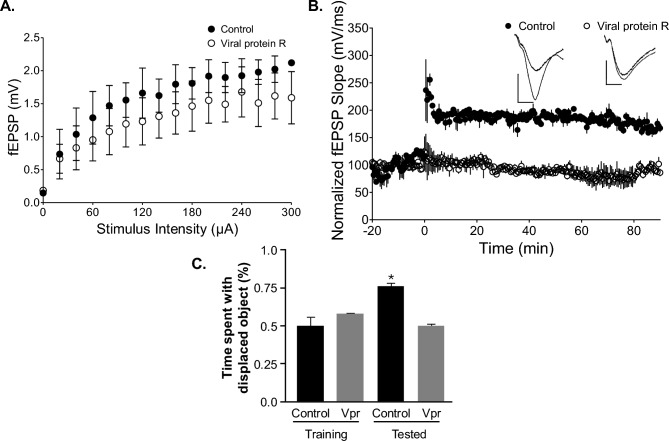
Vpr induces hippocampal synaptic plasticity and impairs spatial memory in an animal model. **A** Hippocampal input/output curves and **B** LTP with representative recordings for slices incubated for 3 h in either 200 ng/ml viral protein R (n = 3) or aCSF (control, n = 2). LTP is expressed as the fEPSP slope normalized to the baseline mean pre-tetanus. Results are shown as mean ± SEM. Scale bars represent 0.5 mV (vertical) and 5 ms (horizontal). **C** Bar graph comparing memory performance between control and Vpr-treated animals during training and testing phases, showing impaired memory in Vpr-treated animals (*p < 0.05).

To evaluate the functional effects of HIV-1 Vpr, hippocampal synaptic plasticity and memory performance were tested in animals treated with or injected with Vpr. The effect of recombinant HIV-1 Vpr protein on hippocampal synaptic function was examined by applying the protein to acute mouse brain slices. Field excitatory postsynaptic potential (fEPSP) recordings at various stimulus levels showed no significant impact of Vpr. Input–output curves are shown in Fig. 5A, indicating normal baseline synaptic transmission. However, Vpr reduced hippocampal long-term potentiation (LTP). LTP measurements are shown in Fig. 5B, suggesting disrupted synaptic plasticity, which is essential for memory formation. These results show that Vpr directly interferes with synaptic plasticity, probably through postsynaptic mechanisms.

Next, we assessed the impact of Vpr on memory in these animals. Mice received stereotaxic injections of Vpr protein (or saline control) bilaterally into the hippocampi, followed by intraperitoneal (IP) injections of saline or Vpr protein. Memory was tested using the object location memory task for spatial memory, 72 h after the stereotaxic injection. During the object location test, mice were trained to find two identical objects in an arena. After three training trials, the mice spent roughly 50% of their time with each object because they were identical. In the test phase, one object (the “displaced object” (DO)) was moved to a new location. If the mouse recalls the original spatial pattern and recognizes that one object has been moved, it will spend more time exploring the displaced object. A significant increase in time spent with the displaced object during the test phase compared to the training phase indicates good spatial memory. Animals injected with Vpr protein spent significantly less time with the DO compared to controls during testing. Memory performance is shown in Fig. 5C, confirming that Vpr impairs spatial memory.

Given the established role of α-Syn in synaptic function, its aggregation in the hippocampus may contribute to the observed memory impairments. α-Syn aggregation is involved in both cognitive and motor problems. In Parkinson’s disease, motor symptoms are prominent initially, and spatial memory deficits develop over time. These findings suggest that Vpr-induced α-Syn aggregation may have diverse effects. The impact depends on the affected brain region and may include both spatial memory and motor functions.

## Discussion

Our research demonstrates that HIV-1 Vpr induces epigenetic modifications at the SNCA antisense promoter, thereby increasing α-synuclein (α-Syn) expression and aggregation in neuronal cells. Vpr demethylates specific CpG sites within this promoter, activating SNCA transcription, which elevates α-Syn levels and promotes aggregation. We observed these effects in cell cultures and human brain tissues from individuals with HIV-associated neurocognitive disorders (HAND), emphasizing Vpr’s critical role in HIV-related neurodegeneration^14,15^. Our data also indicate that α-Syn dysregulation contributes to memory deficits and synaptic dysfunction, potentially explaining the high prevalence of neurocognitive disorders among people living with HIV (PLWH).

A recent study shows that α-synuclein enhances HIV-1 entry and replication in central nervous system (CNS)-resident cells, potentially sustaining viral reservoirs^23^. These findings suggest a feed-forward loop where Vpr-driven α-Syn aggregation both worsens neurodegeneration and promotes HIV-1 infection. Targeting this interaction could lead to new therapies for HAND and related disorders.

Our findings are consistent with previous research showing that Vpr targets epigenetic machinery, including chromatin-modifying proteins and DNA methylation regulators, to counteract host silencing mechanisms^12^. Vpr induces histone demethylation, promotes the degradation of histone deacetylases such as histone deacetylase 1 (HDAC1), HDAC3, and sirtuin 7 (SIRT7), and enhances chromatin accessibility and gene activation^13^. In our chromatin immunoprecipitation sequencing (ChIP-seq) assay for CCCTC-binding factor (CTCF), we observed increased expression of ten-eleven translocation 2 (TET2), which is involved in gene demethylation, along with DNA methyltransferase 3 A (DNMT3A) and DNMT3B, which are responsible for new methylation. These results further support the hypothesis that Vpr may disrupt the methylation and demethylation machinery, thereby promoting α-Syn accumulation. The exact mechanism by which Vpr promotes α-Syn accumulation remains incompletely understood. Vpr may directly activate SNCA transcription by demethylating its promoter, or it could indirectly promote the accumulation of α-syn by modulating the methylation and demethylation machinery that controls α-syn expression. Additionally, Vpr may induce cellular stress and mitochondrial dysfunction, or inhibit autophagy, collectively contributing to α-Syn aggregation, as observed in our previous work^14,15^. We are investigating the relative roles of these pathways. Clarifying these mechanisms will help explain how Vpr-induced α-Syn aggregation causes cognitive impairments, such as spatial memory deficits, and motor problems in HAND and related conditions.

Antisense promoters, especially those found in introns, regulate gene expression in neurodegeneration^24,25^. These promoters trigger long non-coding RNAs (lncRNAs) that control sense genes through chromatin remodeling, DNA methylation, and histone modification^26^. Disruption of these processes contributes to neurodegenerative diseases like Parkinson’s and Alzheimer’s. In our study, we identify the SNCA antisense promoter as essential for regulating α-Syn expression via CpG methylation, linking methylation loss to increased α-Syn expression and aggregation^18–20^. We confirmed that inhibiting Tet with DMOG prevents SNCA antisense promoter demethylation and stops α-Syn from increasing (Fig. 4F). This indicates that Tet activity is required for Vpr-induced demethylation, which in turn increases SNCA-AS expression, revealing a mechanistic link between Vpr action and α-Syn accumulation. However, it remains uncertain whether activating SNCA-AS alone can trigger α-Syn aggregation; upcoming experiments will directly test this by increasing SNCA-AS independently of Vpr using antisense oligonucleotides or clustered regularly interspaced short palindromic repeats (CRISPR) editing. We will also examine whether activation of the SNCA sense promoter occurs concurrently, directly linking SNCA-AS activity to α-Syn expression.

Unresolved questions remain regarding how Vpr affects α-Syn accumulation, particularly the specific level of gene regulation involved. It remains unclear whether Vpr-induced epigenetic changes primarily affect α-Syn gene transcription or protein translation and processing. Additionally, chromatin structural alterations, such as R-loop formation, may contribute to Vpr-mediated regulation of α-Syn. Further clarification of these mechanisms is necessary to fully understand Vpr’s influence.

Studying the SNCA antisense promoter shows how DNA and RNA interact. The antisense promoter located within the first SNCA intron may produce lncRNAs that regulate the sense promoter. Chromatin remodeling, RNA-RNA interactions, and methylation work together to control gene expression^27^. This type of regulation impacts other neurodegenerative genes, such as tumor protein p53 (TP53)^28^ and breast cancer 1 (BRCA1)^29^, as well as viral genomes, including HIV-1^30^. DNA demethylation of antisense promoters can reactivate them, leading to abnormal gene expression and contributing to diseases like cancer, neurodegeneration, and chronic viral infections.

Consistent with this analysis, in brain tissues from controls, PLWH, and individuals with Parkinson’s disease (PD) or Alzheimer’s disease (AD), we observe increased α-Syn levels and more senescence-associated heterochromatic foci (SAHF), linking α-Syn aggregation to cellular aging [^31^ and studies in progress]. Brain tissue analyses show more SAHF-positive and distorted nuclei in HIV cases compared to age-matched controls, matching levels seen in AD and PD (Table S2). This supports the idea that HIV promotes early chromatin condensation and nuclear aging in neurons. α-Syn accumulation may induce neuronal senescence and contribute to cognitive decline in HAND. Increased mammalian target of rapamycin (mTOR) activity in PLWH may enhance cell stress, further increase α-Syn, and connect Vpr-triggered epigenetic changes to aging. Vpr thus causes direct viral damage and accelerates cellular aging, which may help explain persistent motor and cognitive problems in PLWH.

Vpr causes DNA damage and then triggers epigenetic changes that promote disease. Even in non-dividing neurons, Vpr activates DNA damage responses (DDR)^11,32^, which modify chromatin and DNA methylation^12,13^. Our results suggest that Vpr may interact with TET enzymes or their regulators. Inhibiting TET with DMOG halts Vpr-induced demethylation and antisense activation. We need to investigate further how Vpr interacts with these regulators. Although DDR pathways are most recognized in dividing cells, they also alter methylation in neurons, including at the SNCA antisense promoter. This corresponds to patterns observed in other neurodegenerative diseases, in which DNA repair leads to reprogramming of disease genes^33^. Therefore, CpG demethylation at the SNCA antisense promoter may be induced by DNA damage, increase α-Syn levels, and promote aggregation.

Several studies support the hypothesis that CpG demethylation, such as at CpG-2 (intron 1, particularly sites 8 and 12) in SNCA, contributes to α-Syn overexpression, a key factor in neurodegenerative diseases. Matsumoto et al. (2010) emphasized the role of CpG demethylation in regulating α-synuclein expression and aggregation^18^. Similarly, Yang et al. (2017)^19^ showed that CpG demethylation in SNCA intron 1 leads to increased SNCA expression in regions of Parkinson’s disease (PD) brain, including the substantia nigra pars compacta (SNpc), putamen, and cortex. Jowaed et al. (2010)^20^ and Miranda-Morales et al. (2017)^34^ reported significant demethylation of intron 1 in PD patient brain samples, which may explain the elevated expression of SNCA. However, Guhathakurta et al. (2017) suggested that CpG demethylation and α-Syn accumulation are independent of PD development^35^. Despite this, data from Young et al. (2019) and Henderson (2021) confirm significant hypomethylation in genes from the substantia nigra region and blood samples from PD patients, reinforcing the link between methylation loss and PD pathology^36,37^.

To evaluate the relevance of our findings to human neuropathology, we examined brain tissues from both PLWH and SIV-infected monkeys^14^. We found upregulation of α-Syn and demethylation of the antisense promoter, supporting the translational relevance of our in vitro results. Additionally, we detected mitochondrial dysfunction, decreased adenosine triphosphate (ATP) production, impaired mitophagy, and increased reactive oxygen species (ROS) levels in Vpr-treated neurons^15^. These disruptions in cellular energy balance and mitochondrial dynamics, along with endoplasmic reticulum (ER) stress and β-amyloid accumulation, suggest that Vpr-induced α-Syn accumulation impairs cellular function and activates stress pathways implicated in neurodegenerative diseases. Deficits in mitochondrial axonal transport and microtubule stability further implicate α-Syn dysregulation as a key factor in neuronal damage and potential cognitive and motor impairments^15^.

In vivo, Vpr-treated mice showed cognitive impairments, including deficits in spatial memory and long-term potentiation (LTP), aligning with the established role of α-Syn in cognitive decline associated with HAND. Structural changes, such as brain atrophy, were also observed in PLWH^38^, further linking molecular alterations in SNCA regulation to neurodegeneration. These findings from animal models, combined with human brain tissue data, highlight the significance of Vpr-induced epigenetic modulation of the SNCA antisense promoter in promoting neurodegenerative processes in HAND. This research clarifies the complex relationship between viral factors, epigenetic modifications, and neurodegenerative disease progression, laying the foundation for developing new therapeutic strategies for HAND and related disorders.

In addition to HIV-1 Vpr, other viral proteins, such as those from severe acute respiratory syndrome coronavirus 2 (SARS-CoV-2), have been shown to interact with α-Syn, thereby causing Lewy body-like pathology in vitro^39^. This indicates that viral proteins can affect α-Syn behavior, potentially contributing to neurodegenerative processes.

These findings have significant clinical implications for the management of HAND in PLWH. Identifying specific epigenetic mechanisms responsible for α-Syn accumulation opens new avenues for therapy. Targeting the Tet-mediated demethylation pathway or antisense promoter activity may decrease α-Syn aggregation and neurodegeneration. Additionally, measuring α-Syn levels or methylation status at the SNCA antisense promoter could serve as biomarkers for early detection of neurocognitive decline in PLWH, enabling prompt intervention. Future research with larger cohorts of PLWH will be needed to confirm these potential biomarkers and therapeutic targets.

In summary, this study provides evidence that HIV-1 Vpr causes epigenetic changes at the SNCA antisense promoter, leading to α-Syn dysregulation and aggregation. These are key factors in HIV-associated neurodegeneration. The similarities between these findings and the known role of epigenetic modifications in synucleinopathies suggest that HIV-1 infection may share mechanisms with diseases like Parkinson’s disease. This research sheds light on how Vpr-induced epigenetic changes may affect hippocampal synaptic plasticity, spatial memory, and motor function in PLWH, potentially explaining motor impairments such as gait disturbances and balance issues. Further research on these epigenetic changes in larger groups of PLWH may help identify new biomarkers for early detection and develop new treatment targets, leading to improved management of HIV-related cognitive issues and neurodegeneration.

Finally, examining the SNCA antisense promoter shows how viral proteins manipulate host epigenetic machinery to promote neurodegeneration. Understanding these interactions will identify therapeutic targets for reducing HAND and possibly other α-synucleinopathies.

## Supplementary Information

Supplementary Information.

## Data Availability

All processed data are included in this manuscript. Raw data, further information, or reagents contained within the manuscript are available upon request from the corresponding author, Maryline Santerre, [maryline.santerre@temple.edu].

## Declarations

### AI Assistance

The manuscript was enhanced with the assistance of AI, specifically ChatGPT, to improve clarity, flow, and readability. AI was used exclusively for text editing and refinement, with no involvement in data analysis.
