# Supplementary material for: HIV Vpr induces demethylation of the SNCA antisense promoter, leading to neurocognitive impairment
Source: Sci Rep. 2026 Jan 23;16:6078. doi: 10.1038/s41598-026-35691-3 (PMC12902034; doi:10.1038/s41598-026-35691-3)
Supplement: Supplementary file 1 — Supplementary Information. [file 41598_2026_35691_MOESM1_ESM.pdf]

SUPPLEMENT

Patient ID	Age	Normal=0<br>Pre-HAART=1<br>Post-HAART=2	Neuropathology
7101958083	46	0	Normal
7101198377	34	0	Normal
OBB351-1	33	0	Normal
OBB119-1	45	0	Normal
OBB349-2	50	0	Normal
OBB173-1	40	0	Normal
OBB154-1	35	0	Normal
OBB227-1	33	0	Normal
OBB325-1	47	0	Normal
OBB376-1	38	0	Normal
OBB329-2	46	0	Normal
OBB167-1	52	1	Alzheimer type 2 gliosis
OBB238-1	33	1	Cerebral atrophy
OBB003-3	36	1	Normal
OBB305-1	40	1	Microglial nodules
OBB120-3	40	1	Focal ischemic neuronal necrosis and Bergmann gliosis
OBB083-1	50	1	Bergman’s gliosis, Acute neuronal changes
OBB110-1	44	1	Meningistis, brain hernia, cerebral edema
OBB192-2	40	1	Multiple lesions with gliosis and necrosis
OBB297-2	39	1	HIV encephalitis, microglial nodules
OBB341-1	48	1	HIV encephalitis
OBB151-1	48	1	Chronic meningoencephalitis
4049	44	2	Other non-infectious pathology
7102066884	50	2	Other non-infectious pathology
4002	35	2	Alzheimer type 2 gliosis, other non-infectious pathology
6078	37	2	Alzheimer type 2 gliosis, aseptic leptomeningitis
6037	41	2	Minimal non-diagnostic abnormalities
7100696768	42	2	Microglial nodule encephalitis
7200116866	35	2	Alzheimer type 2 gliosis, focal infarct
MHBB500	47	2	HIV encephalitis
10162	42	2	Microglial nodule encephalitis
CE102	48	2	HIV encephalitis
CA235	46	2	Microglial nodule encephalitis

Table S1. Case-level metadata for human brain samples used in Figure 4 analyses. De-identified repository ID, age (years), HIV/ART group (0 = HIV– control; 1 = HIV+ pre-ART; 2 = HIV+ post-ART), and repository neuropathology summary are shown. n = 33 male donors (HIV–, n = 11; HIV+ pre-ART, n = 11; HIV+ post-ART, n = 11); age range 33–52 years. Tissues were obtained from the National NeuroAIDS Tissue Consortium (NNTC). Neuropathology terms reflect repository summaries. Only age, sex, HIV/ART status, and brief neuropathology were available; broader comorbidity, ART regimen, and viral load data were not provided.

PID	group	age	gender	Brain area	% nuclei with SAHF	% deshaped nuclei
1	Normal	35	M	Temporal	10.41	1.36
2	Normal	48	M	Temporal	4.86	7.32
3	Normal	50	M	Temporal	48.39	4.43
4	HIV	34	M	Temporal	48.25	13.99
5	HIV	36	M	Temporal	27.58	13.68
6	HIV	48	M	Temporal	18.08	52.13
7	Normal	45	M	Frontal	20.29	4.35
8	Normal	46	M	Frontal	25.43	0.87
9	HIV	46	M	Frontal	49.65	1.42
10	HIV	47	M	Frontal	45.88	16.67
11	Normal	45	M	Hippocampus	7.93	3.66
12	Normal	46	M	Hippocampus	25.64	2.56
13	HIV	46	M	Hippocampus	52.57	0.57
14	NFT	63	M	Temporal	65.43	4.86
15	AD	69	M	Temporal	70.71	7.86

Table S2. Case-level senescence and nuclear morphology metrics in human brain tissue. De-identified male donors (n=15; ages 34-69) across diagnostic groups (Normal, HIV, NFT, AD) and brain regions (temporal, frontal, hippocampus). Columns list patient ID (PID), group, age, gender, brain area, percent nuclei with senescence-associated heterochromatin foci (% SAHF), and percent de-shaped nuclei. SAHF were defined as DAPI-dense, punctate heterochromatin foci within nuclei; de-shaped nuclei were defined by irregular nuclear contours (non-elliptical morphology). For each case/region, ≥10 non-overlapping fields were scored by a blinded rater, and values are reported as the mean percentage per case. These data provide an orthogonal readout of cellular stress/senescence in the same regions analyzed for SNCA methylation and α-synuclein, supporting the link between Vpr-associated stress, epigenetic remodeling, and neurodegeneration. Abbreviations: SAHF, senescence-associated heterochromatin foci; NFT, neurofibrillary tangles; AD, Alzheimer's disease.
